# Liangxue Jiedu Formula Improves Psoriasis and Dyslipidemia Comorbidity *via* PI3K/Akt/mTOR Pathway

**DOI:** 10.3389/fphar.2021.591608

**Published:** 2021-03-03

**Authors:** Xinran Xie, Lei Zhang, Xue Li, Weihong Liu, Ping Wang, Yan Lin, Xuyang Han, Ping Li

**Affiliations:** ^1^ Beijing Hospital of Traditional Chinese Medicine, Capital Medical University, Beijing, China; ^2^ Beijing Institute of Traditional Chinese Medicine, Beijing, China; ^3^ Dongfang Hospital, Beijing University of Chinese Medicine, Beijing, China

**Keywords:** psoriasis, dyslipidemia, PI3K/Akt/mTOR pathway, ApoE−/− mice, imiquimod (IMQ), traditional Chinese medicine

## Abstract

The pathological mechanism of psoriasis and dyslipidemia comorbidity is unclear, and there are few reports on therapy. By establishing an animal model of ApoE^−/−^ mice induced by imiquimod (IMQ), we explored the effects of Liangxue Jiedu formula (LXJDF), a traditional Chinese herb medicine, on psoriasis and dyslipidemia comorbidity through PI3K/Akt/mTOR pathway. The experiment was divided into a control group, a model group, an LXJDF high-dose group, an LXJDF low-dose group, and a positive drug (atorvastatin) group. Each group of mice was given continuous oral administration once a day. After 3 weeks, the mice dorsal skins were smeared with 62.5 mg of 5% IMQ cream for five consecutive days and continued to be given the corresponding drugs. We observed the effects of LXJDF on skin lesion changes, PASI score, pathological characteristics, blood lipid levels (TC, TG, LDL, HDL, and oxLDL), liver pathology, inflammatory factors in the skin, and the protein expression of PI3K/Akt/mTOR pathway in both the skin and liver. The results showed that LXJDF could significantly improve the psoriasiform skin lesions of IMQ-induced ApoE^−/−^ mice, including the reduction of PASI, thinning of epidermal thickness, inhibition of hyperkeratosis and parakeratosis, and inflammatory infiltration in the dermis, and reduce lipid accumulation in the epidermal. LXJDF could regulate blood lipid levels, reduce liver inflammation, and protect the liver. LXJDF could significantly decrease the gene expressions of inflammatory factors IL-17A, IL-23, IL-6, and TNF-α in the skin. LXJDF showed specific inhibition of PI3K, Akt, mTOR protein, and its phosphorylation expressions. In conclusion, LXJDF exerts an intervention effect on psoriasis and dyslipidemia comorbidity *via* PI3K/Akt/mTOR and its phosphorylation pathway.

## Introduction

Psoriasis is an immune-mediated chronic inflammatory skin disease. Since 2011, Boehncke et al. put forward the concept of “psoriatic march,” when psoriasis develops the severity phase causing systemic inflammation, which causes insulin resistance and triggers endothelial cell dysfunction, then leading to atherosclerosis and cardiovascular disease (CVD) (Boehncke et al., 2011; Coumbe et al., 2014). Epidemiological data indicate that the incidence of potential CVD in patients with psoriasis is 25% higher than that in non-psoriasis patients (Aldona et al., 2013). Some scholars even proposed that psoriasis is an independent risk factor for myocardial infarction (MI) (El-Mongy et al., 2010; Dattilo et al., 2018). The average life expectancy of patients with severe psoriasis is shortened by 5 years. Psoriasis patients accompanied by CVD are the main reason for death, making psoriasis a complex life-threatening disease (Mehta et al., 2011).

Recently, there were a large number of research reports on psoriasis combined with CVD. The most common comorbidity was dyslipidemia, mainly manifested as total cholesterol (TC), and triglyceride (TG) and low-density lipoprotein cholesterol (LDL-C) levels increased, and high-density lipoprotein cholesterol (HDL-C) components decreased (Coumbe et al., 2014). Meanwhile, patients’ skin lesions also showed lipid metabolism abnormality (Tekin et al., 2007; Varshney and Saini, 2018). Studies have suggested that systemic anti-inflammatory therapy can improve psoriasis and its comorbidities simultaneously. However, the literature on biological agent (such as TNF-α antagonists) treatment was inconsistent. Some accepted their improvement; others considered those that may induce and aggravate comorbidities (Ryan et al., 2011). Specialists have realized that psoriasis therapy aims to eliminate skin lesions and block the “psoriasis march,” and curb the deterioration of metabolism syndrome (MS) and CVD. Dyslipidemia is a significant risk factor for CVD’s occurrence and development, including MS, MI, and atherosclerosis, and affects the course and prognosis of these diseases. Therefore, it is of considerable significance to improve the quality of life and survival rate of psoriasis patients accompanied by dyslipidemia.

Psoriasis is an autoimmune disease mediated mainly by Th1/Th17/Th22 cells. These T cells and their secreted inflammatory cytokines, such as TNF-α, IL-6, IL-17, and IL-22, interacted with dendritic cells (DCs), keratinocytes (KCs), and vascular endothelial cells and formed the complex network, which plays an essential role in the development of psoriasis (Lowes et al., 2014). Dyslipidemia causes the imbalance of lipid composition and abnormal lipoprotein levels in the blood. The increase of intracellular cholesterol promotes the secretion of inflammatory cytokines such as IL-17A, TNF-α, and IL-6. Otherwise, inflammatory cytokines and the corresponding pathway activation can also affect the biosynthesis of cholesterol and fatty acids (Li et al., 2005).

The phosphatidylinositol-3-kinase (PI3K), protein kinase B (PKB/Akt), and mammalian target of rapamycin (mTOR) pathway play a vital role in cell survival and proliferation, cell apoptosis, autophagy, and metabolic regulation. Besides, it has an extensive and particular function in innate immune cells, including neutrophile granulocyte, mast cells, monocytes, macrophages, and DCs, which are all related to the immune pathogenesis of psoriasis (Huang et al., 2014). The activation of the PI3K/Akt/mTOR pathway promotes the secretion of inflammatory cytokines such as IL-17A, TNF-α, and IL-1β (Buerger, 2018). The relative proteins of the PI3K/Akt/mTOR pathway have a potential therapeutic target for psoriasis, such as everolimus (rapamycin derivatives) (Frigerio et al., 2007; Bürger et al., 2017).

Liangxue Jiedu formula (LXJDF) is a traditional Chinese medicine (TCM) compound for the treatment of psoriasis, which can effectively improve psoriasis patients’ skin lesions and regulate the glycolipids metabolism as well (Liu et al., 2009; Liu et al., 2010). LXJDF could significantly improve imiquimod- (IMQ-) induced mice psoriatic skin lesions, reduce the PASI score, and inhibit epidermal cell hyperplasia, parakeratosis, and inflammatory cell infiltration. It also could decrease IL-23/Th-17 axis-related cytokine expressions and RORγt (Th17 cell differentiation transcription factor) mRNA expression (Zhao et al., 2015).

To study the pathogenesis and pharmacotherapeutics of psoriasis and dyslipidemia comorbidity, we established a composite animal model by applying IMQ on the dorsal skin of ApoE^−/−^ mice. Preliminary evaluation of the model confirmed that it could cause psoriasiform lesions by IL-23/Th17 axis activation and show the pathological characteristics of dyslipidemia (Xie et al., 2017). Therefore, we observed the effect of LXJDF on the IMQ-induced ApoE^−/−^ mice model to study the intervention of LXJDF on psoriasis and dyslipidemia comorbidity via regulating the PI3K/Akt/mTOR pathway.

## Materials and Methods

### Preparation of LXJDF

Liangxue Jiedu formula (LXJDF), which contains thirteen herbs (Table 1), is provided by TCM Pharmacy of Beijing Hospital of Traditional Chinese Medicine affiliated to Capital Medical University. The herbs were authenticated by Dr. Tieying Wang (Beijing Xinglin Pharmaceutical Co., Ltd.) following standard protocols of the Chinese Pharmacopoeia (Version 2015). All the herbaria are stored in the specific Herbarium Room of Beijing Xinglin Pharmaceutical Co., Ltd. 498 g of crude drugs of LXJDF was soaked for 60 min in 4,000 ml of pure water (eight times volume) and decocted using reflux extraction methods for 40 min. Then filter out drug liquid and decocted in 3,000 ml pure water (six times volume) for 20 min. The two-drug liquid was combined and concentrated to the experimental dosage. The final dosage of LXJDF-high dose (H) was 42.6 g/kg·bw, and LXJDF-low dose (L) was 21.3 g/kg·bw (equal to human clinical equivalent-effective dose) (Xu et al., 2006).

**TABLE 1:** Components of LXJDF.

Chinese name	Scientific name	Used part	Amount (g)	Lot no.	Place of origin	Company	Voucher numbers
Zi cao	*Arnebia euchroma* (Royle ex Benth.) I.M.Johnst	Root	10	18,031,003	Xinjiang, China	Beijing Xinglin Pharmaceutical Co., Ltd.	*Arnebia euchroma* (Royle ex Benth.) I.M.Johnst., root (No. 18031003-Wang)
Chi shao	*Paeonia lactiflora* Pall	Root	10	18,011,401	Inner Mongolia, China	Beijing Xinglin Pharmaceutical Co., Ltd.	*Paeonia lactiflora* Pall., root (No. 18011401-Wang)
Sheng di huang	*Rehmannia glutinosa*	Root and rhizome	15	18,011,602	Henan, China	Beijing Xinglin Pharmaceutical Co., Ltd.	*Rehmannia glutinosa.*, root and rhizome (No. 18011602-Wang)
Dan pi	*Paeonia × suffruticosa* Andrews	Velamen	10	18,020,901	Anhui, China	Beijing Xinglin Pharmaceutical Co., Ltd.	*Paeonia × suffruticosa* Andrews*.,* velamen (No. 18020901-Wang)
Jin yin hua	*Lonicera japonica* Thunb	Dry flower	15	18,011,701	Shandong, China	Beijing Xinglin Pharmaceutical Co., Ltd.	*Lonicera japonica* Thunb*.*, dry flower (No. 18011701-Wang)
Bai hua she she cao	*Scleromitrion diffusum* (Willd.) R.J.Wang	Whole grass	30	18,021,703	Guangdong, China	Beijing Xinglin Pharmaceutical Co., Ltd.	*Scleromitrion diffusum* (Willd.) R.J.Wang., whole grass (No. 18021703-Wang)
Tu fu ling	*Smilax glabra* Roxb	Rhizome	15	18,021,501	Guangdong, China	Beijing Xinglin Pharmaceutical Co., Ltd.	*Smilax glabra* Roxb., rhizome (No. 18021501-Wang)
Huai hua	*Styphnolobium japonicum* (L.) Schott	Dry flower	10	18,011,703	Shaanxi, China	Beijing Xinglin Pharmaceutical Co., Ltd.	*Styphnolobium japonicum* (L.) Schott., dry flower (No. 18011703-Wang)
Yin chen	*Artemisia capillaris* Thunb	Dry aboveground part	15	18,010,901	Shaanxi, China	Beijing Xinglin Pharmaceutical Co., Ltd.	*Artemisia capillaris* Thunb*.*, dry aboveground part (No. 18010901-Wang)
Pu huang	*Typha orientalis* C.Presl	Pollen	10	17,090,235	Jiangsu, China	Beijing Xinglin Pharmaceutical Co., Ltd.	*Typha orientalis* C.Presl., pollen (No. 17090235-Wang)
Qing dai	*Isatis tinctoria L*	Processed dry powder of leaves	6	18,020,106	Fujian, China	Beijing Xinglin Pharmaceutical Co., Ltd.	*Typha orientalis* C.Presl., processed dry powder of leaves (No. 18020106-Wang)
Chen pi	*Citrus × aurantium* L.	Mature peel	10	18,021,085	Sichuan, China	Beijing Xinglin Pharmaceutical Co., Ltd.	*Citrus × aurantium* L.*,* mature peel (No. 18021085-Wang)
Shan zha	*Crataegus pinnatifida* Bunge	Mature fruit	10	18,022,702	Liaoning, China	Beijing Xinglin Pharmaceutical Co., Ltd.	*Crataegus pinnatifida* Bunge., mature fruit (No. 18022702-Wang)

### Reagents

Imiquimod cream was purchased from Sichuan Mingxin Pharmaceutical Co., Ltd. (Sichuan, China). Atorvastatin was purchased from Pfizer Pharmaceutical Co., Ltd. (Liaoning, China). Hematoxylin and eosin (H&E) for HE staining were purchased from Leica (Wetzlar, Germany). OpalTM 4-Color Manual IHC Kit and Opal 620 Fluorophore were purchased from PerkinElmer (Waltham, MA, United States). Oxidized low-density lipoprotein (oxLDL) kit was purchased from Nanjing Jiangcheng Bioengineering Institute (Jiangsu, China). Antibodies for PCNA, PI3Kp110α, Akt, Phospho-Akt (Thr308), Phospho-Akt (Ser473), and Phospho-mTOR (Ser2448) were purchased from Cell Signaling Technology (Danvers, MA, United States). Antibodies for GAPDH, Ki67, loricrin, LOX-1, CD3, CD4, CD11c, F4/80, PI3Kp85α, mTOR, Phospho-mTOR (S2481), Goat Anti-Rat IgG H&L (HRP), and Rabbit Anti-Armenian hamster IgG H&L (HRP) were purchased from Abcam (Cambridge, UK). PierceTM bicinchoninic acid (BCA) protein assay kit was purchased from Thermo Scientific (Cleveland, OH, United States). TRIzol reagent and all primers were purchased from Invitrogen (Waltham, MA, United States). PrimeScript™ RT reagent kit with gDNA Eraser and TB Green® Premix Ex Taq™ II (Tli RNaseH Plus), ROX plus, were purchased from Takara BioMed Co., Ltd. (Liaoning, China). ECL Ultra Western HRP Kit was purchased from Merck Millipore (Darmstadt, Germany).

### UPLC-MS/MS Analysis of LXJDF

An ultrahigh performance liquid chromatography-tandem mass spectrometry (UPLC-MS/MS) spectrometer with a HESI-II probe was employed to analyze LXJDF. DIONEX Ultimate 3,000 ultra-high-performance liquid chromatography and Thermo Hypersil Gold C18 column (3 μm × 2.1 mm × 100 mm) were applied. The mobile phase was composed of A (water, 2 mmoL/L ammonium formate, and 0.1% formic acid, v/v) and B (acetonitrile) with gradient elution. The flow rate is set to 0.25 ml/min and the column temperature is maintained at 45°C. The injection volume is 5 μl. Thermo Q EXACTIVE mass spectrometer was applied. The positive and negative HESI-II spray voltages were 3.5 kV, and the heated vaporizer temperature was 350°C. Both the sheath gas and the auxiliary gas were nitrogen. The capillary temperature was 320°C, and S-lens RF was 50. The content of the main compounds of LXJDF (prepared in three batched) was detected with the standard as a reference using UPLC.

### Animal Model and Groups

Male ApoE^−/−^mice (C57BL/6J strain) and C57BL/6J mice aged 28 weeks were purchased from Beijing Vital River Experimental Animal Technology Co., Ltd. (Beijing, China). The animal experiment was strictly handled according to *Guide for the Care and Use of Laboratory Animals* (NIH Publication No. 85-23,1996). Experimental procedures were managed according to the local ethics committee.

**FIGURE 1:**
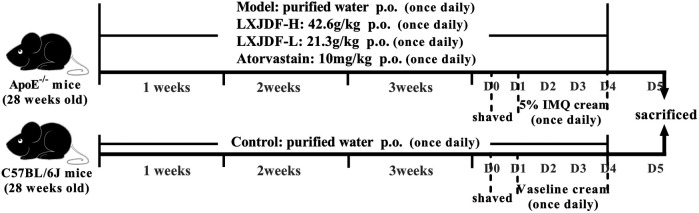
Experimental procedure of intervention study of LXJDF. The experiment was divided into five groups: control, model, LXJDF-H, LXJDF-L, and atorvastatin, continuous oral administration once daily. After 3 weeks, 62.5 mg of 5% IMQ cream was applied for five consecutive days, and all mice were given corresponding drugs continuously.

Forty ApoE^−/−^mice were randomly divided into four groups: model, LXJDF-H (42.6 g/kg), LXJDF-L (21.3 g/kg), and atorvastatin (10 mg/kg, Pfizer) groups. Ten C57BL/6J wild-type (WT) mice were in the control group. All mice were gavaged with the drugs once a day with a normal diet. The mice in the control and model group were given an equal amount of purified water. After 3 weeks of administration, all 50 mice were anesthetized with pentobarbital sodium intraperitoneal injection (80 mg/kg) and collected in a single cage after adjacent hair removal, and the next day mice dorsal skin received a topical dose of 62.5 mg of 5% IMQ cream for five consecutive days, respectively. Control mice were smeared with Vaseline (Xie et al., 2017) (Figure 1). We observed the skin lesions of the mice every day and photographed them. According to the PASI scoring standard, give the mice erythema, scales, and thickness 0–4 points on the skin lesions, respectively, and add the three integrals to get the total integrals, draw the average of the integrals of the mice in each group, and then draw the trend line of the integral lesions to observe the changes of the lesions of the mice in each group.

### Histology

Paraffin-embedded skin or liver samples were sectioned and stained with hematoxylin and eosin (HE) staining. Histopathological changes were scanned in the Aperio CS2 Leica scanner (Leica, Germany). Epidermal thickness was measured using the software Image Scope^TM^ (Aperio Technologies).

### Immunofluorescence Staining

Paraffin-embedded skin samples were cut into 3 μm tissue sections. The detailed steps were performed according to the OpalTM 4-Color Manual IHC Kit instructions. All slides were dewaxed with xylene and rehydrated through a graded series of ethanol solutions. Then, they performed antigen retrieval using microwave treatment and blocking. After incubation with the primary antibody and corresponding secondary-HRP antibody, the slides were incubated with Opal Fluorophore working solution. Then the above steps were repeated, and the corresponding antibodies were incubated in sequence. The primary antibody dilution concentration and reaction conditions are listed in Supplementary Table S1. DAPI was applied for counterstain. The sections were observed by laser scanning confocal microscope (Zeiss LSM710, Germany) and taken photographs under the excitation wavelength of 405, 520, 570, 620, and 690 nm, respectively, then merged images *in situ*.

### Serum Lipids Level Examinations

The concentrations of TC, TG, LDL, and HDL in serum were measured using an automatic biochemical analyzer (Roche P800, Switzerland). The oxidized low-density lipoprotein (oxLDL) kit detected the level of oxLDL in serum according to the reagent instructions.

### Real-Time Quantitative Polymerase Chain Reaction (qPCR)

Total RNA was extracted using the RNeasy Mini Kit. cDNA reverse transcription was performed using PrimeScript™ RT reagent Kit with gDNA Eraser. qPCR was performed in triplicate using TB Green® Premix Ex Taq™ II (Tli RNaseH Plus), ROX plus on 7,500 Real-Time PCR System (Applied Biosystems, Thermo Fisher, United States). The reaction conditions were started at 95°C for 30 s, followed by 45 cycles at 95°C for 5 s and 60°C for 40 s. Gene expression levels were normalized to β-actin using 2^−ΔΔCt^ method. The primers for qPCR are shown in Supplementary Table S2.

### Western Blot Analysis

Total protein was extracted using protein lysis buffer (RIPA:PMSF = 100:1) and quantified by the Pierce^TM^ BCA protein assay kit. Samples with an equal amount of proteins and denaturated at 95°C for 5 min. Protein bands were separated using 12% SDS-PAGE and transferred to polyvinylidene fluoride (PVDF) membranes. After blocking in 5% skim milk in TBST (containing 0.1% Tween 20) at 37°C for 1 h, membranes were incubated with primary antibodies at 4°C overnight and then incubated with corresponding peroxidase-conjugated IgG antibodies at 37°C for 1 h. The blots were detected using electrochemiluminescence (ECL) reagent film exposure for 3–5 min, developing 2 min, and fuser. The band integrated density was quantified by ImageJ software. GAPDH antibody was used to confirm the equal amount of protein loading in each lane.

### Statistical Analysis

The experimental data were expressed as “mean ± SEM.” The statistical analyses were performed using a one-way analysis of variance (ANOVA) between groups analyzed by SPSS 22.0 analysis software. The homogeneity of variance was tested by LSD; the heterogeneity of variance was tested by the nonparametric Kruskal–Wallis test. *p < 0.05 and **p < 0.01 mean statistical significance.

## Results

### Identification of Major Components of LXJDF

**FIGURE 2:**
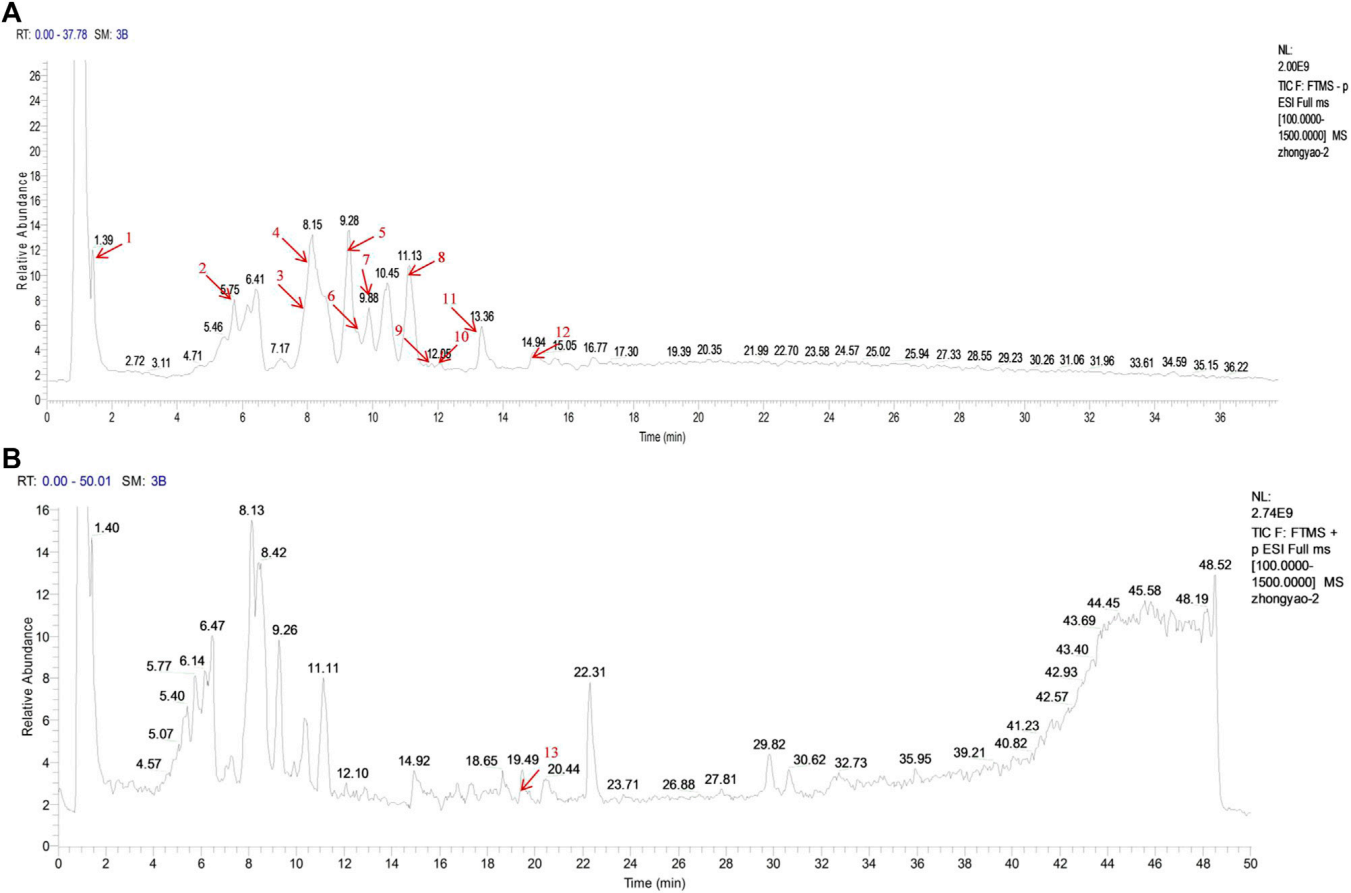
Identification of major components of Liangxue Jiedu formula (LXJDF) using UPLC-MS/MS. The positive **(A)** and negative **(B)** ion chromatograms of LXJDF were shown as indicated. The red arrow marks the retention time (t_*R*_/min) of each major component, and the serial number corresponds to Table 2.

We used UPLC-MS/MS to determine the major components of LXJDF. The total positive and negative ion chromatograms of LXJDF are shown in Figure 2. The exact mass number and secondary level fragment number and identification of possible compound names of the main ingredients of LXJDF are listed in Table 2. The chromatograms and content determination of the main compound are shown in Supplementary Figure S1 and listed in Supplementary Table S3.

**TABLE 2:** Information of main compounds of LXJDF.

No.	*t*_*R*_/min	Ion	Exact mass number (m/z)	Secondary-level fragments (m/z)	Chemical formula	Compounds name	Source
1	1.45	[M-H]	361.1140	97.0284	C15H22O10	Catalpol	*Rehmannia glutinosa*
2	5.75	[M-H]	353.0878	191.0544	C16H18O9	Chlorogenic acid	*Lonicera japonica* Thunb*.* and *Crataegus pinnatifida* Bunge
3	8.07	[M-H]	165.0557	75.0078	C9H10O3	Paeonol	*Paeonia × suffruticosa* Andrews
4	8.11	[M-H]	479.1559	121.0382	C23H28O11	Paeoniflorin	*Paeonia lactiflora* Pall*.* and *Paeonia × suffruticosa* Andrews
5	9.17	[M-H]	769.2197	299.0171	C34H42O20	Typhaneoside	*Typha orientalis* C.Presl
6	9.62	[M-H]	463.0882	300.0220	C21H20O12	Hyperoside	*Crataegus pinnatifida* Bunge
7	9.92	[M-H]	449.1089	151.0024	C21H22O11	Astilbin	*Smilax glabra* Roxb
8	11.09	[M-H]	609.1825	301.0689	C28H34O15	Hesperidin	*Citrus × aurantium* L.
9	11.89	[M-H]	287.0925	133.0283	C16H16O5	Shikonin	*Arnebia euchroma* (Royle ex Benth.) I.M.Johnst
10	12.01	[M-H]	447.0932	285.0378	C21H20O11	Galuteolin	*Lonicera japonica* Thunb
11	13.32	[M-H]	301.0354	151.0023	C15H10O7	Quercetin	*Artemisia capillaris* Thunb*.*, *Styphnolobium japonicum* (L.) Schott, and *Scleromitrion diffusum* (Willd.) R.J.Wang
12	14.86	[M-H]	227.0714	185.0592	C14H12O3	Resveratrol	*Smilax glabra* Roxb
13	19.18	[M+H]	263.0815	219.1724	C16H10N2O2	Indirubin	*Isatis tinctoria* L*.*

### Effects of LXJDF on Psoriasiform Skin Lesions in IMQ-Induced ApoE^−/−^ Mice

**FIGURE 3:**
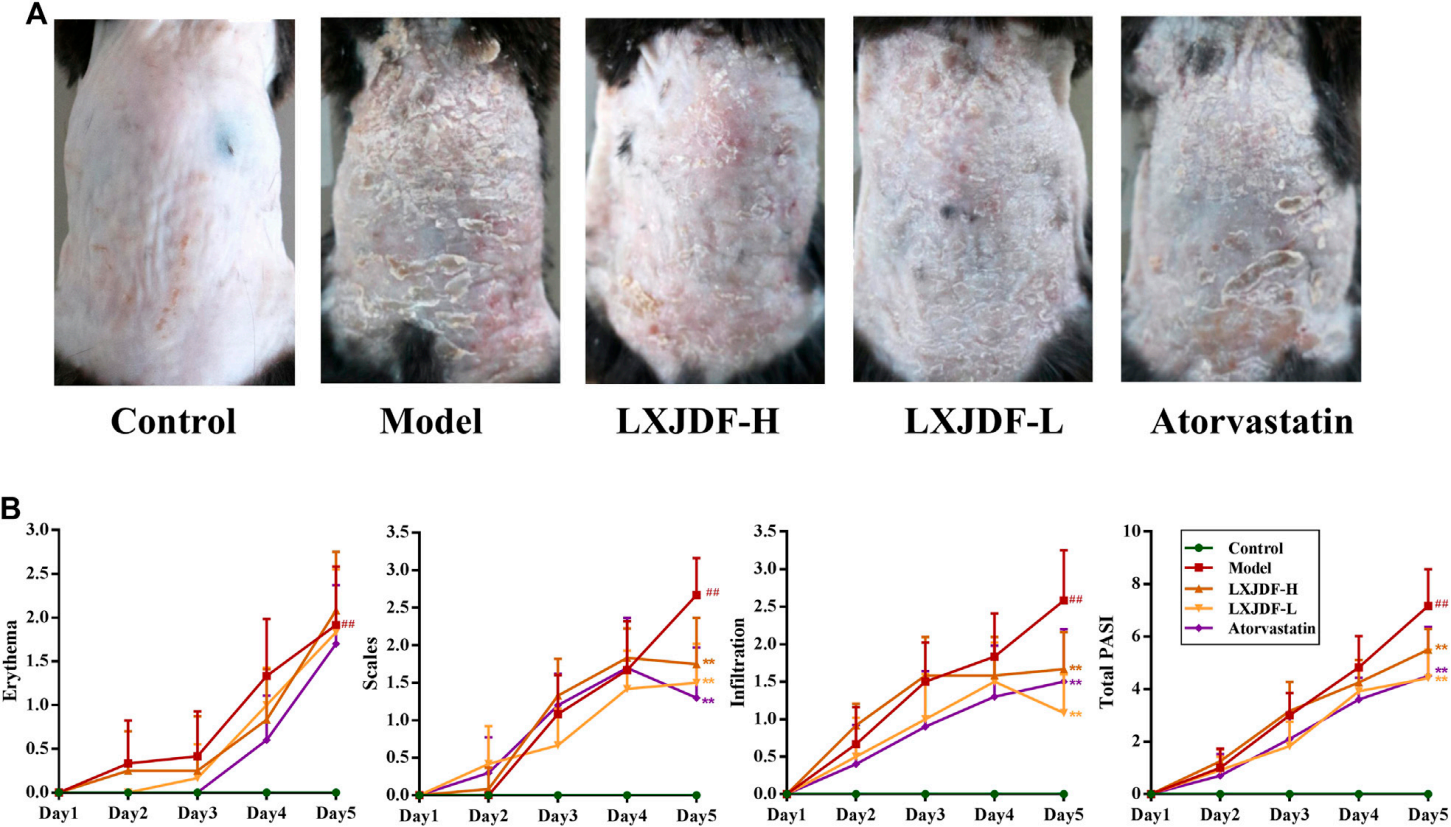
Effects of LXJDF on psoriasiform skin lesions in IMQ-induced ApoE^−/−^ mice. **(A)** Photographs of psoriasiform skin in control, model, LXJDF-H, LXJDF-L, and atorvastatin groups (*n* = 10). **(B)** Single (erythema, scaling, and thickness) and cumulative scores of PASI in control, model, LXJDF-H, LXJDF-L, and atorvastatin groups (*n* = 10). Results are shown as the mean ± SEM. ^#^ *p* < 0.05 and ^##^ *p* < 0.01 vs. control group; ^*^ *p* < 0.05 and ^**^ *p* < 0.01 vs. model group.

To evaluate the effects of LXJDF on pathological features of skin lesions in IMQ-induced ApoE^−/−^ mice psoriasiform skin lesions, we assessed erythema and scaling and infiltrating and skin thicknesses. At the end of the 5-day treatment period, IMQ-induced ApoE^−/−^ mice dorsal skin showed hypertrophic and infiltrated, with a lot of plaque-like scales, red skin color, and a little bleeding. LXJDF-treated mice skin erythema and scales were significantly reduced, as well as infiltration (Figure 3A). Regarding the clinical PASI scoring standard, the mice were scored from the three aspects of erythema, scaling, and infiltration and added up together as a total score. The erythema, scales, infiltration, and total score of IMQ-induced ApoE^−/−^ mice continued to increase with the number of days. Since the third day, the erythema, scales, infiltration, and total score of the LXJDF group were significantly lower than the model. From the statistical results on the fifth day, the LXJDF group had significant differences in scales, infiltration, and total score but had little effect on erythema (Figure 3B).

### Effects of LXJDF on Psoriasiform Skin Lesions Pathological Characteristics in IMQ-Induced ApoE^−/−^ Mice

**FIGURE 4:**
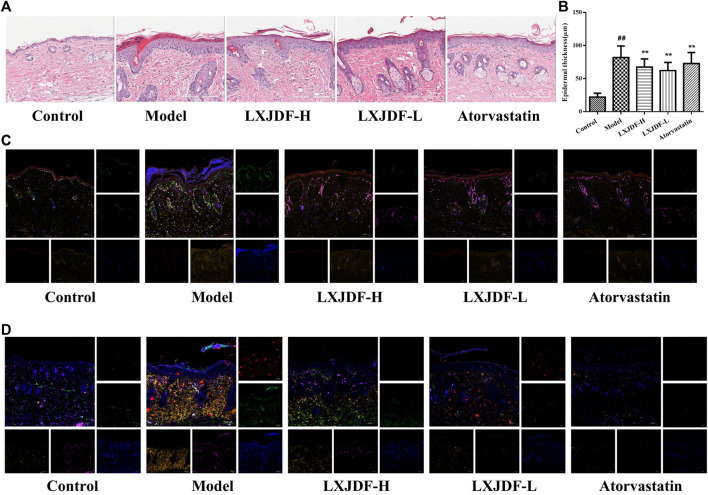
Effects of LXJDF on psoriasiform skin lesions pathological characteristics in IMQ-induced ApoE^−/−^ mice. **(A)** Hematoxylin and eosin (H&E) staining of the skin in control, model, LXJDF-H, LXJDF-L, and atorvastatin groups. Scale bar = 200 μm. **(B)** Microscopic quantification of epidermal thickness in control, model, LXJDF-H, LXJDF-L, and atorvastatin groups. Results are shown as the mean ± SEM. ^#^ *p* < 0.05 and ^##^ *p* < 0.01 vs. control group; *p < 0.05 and **p < 0.01 vs. model group. **(C)** Immunofluorescence staining for loricrin (red), PCNA (green), Ki67 (purple), LOX-1 (gold), and DAPI (blue) in skin of control, model, LXJDF-H, LXJDF-L, and atorvastatin groups. Scale bar = 100 μm. **(D)** Immunofluorescence staining for CD3 (red), CD4 (green), CD11c (purple), F4/80 (gold), and DAPI (blue) in skin of control, model, LXJDF-H, LXJDF-L, and atorvastatin groups. Scale bar = 100 μm.

Compared with the control group, the epidermis of model mice revealed keratinocyte hyperplasia (acanthosis) and parakeratosis. There were a large number of inflammatory infiltrations in the dermal and scattered inflammatory cells in the epidermis. The epidermis of mice in the LXJDF-H and LXJDF-L groups showed that parakeratosis reduced, epidermal thickness became thinner, and inflammatory infiltration alleviated. The epidermal thickness was measured by the image analysis software. LXJDF could significantly reduce the epidermis thickness induced by IMQ (*p* < 0.01). Compared with the model group, the skin tissue epidermis thickening of mice in each dose group of LXJDF was significantly reduced (*p* < 0.01). Atorvastatin had the effect of reducing epidermal thickness and improving hyperplasia and parakeratosis (Figures 4A,B).

To observe the pathological characteristics of psoriasis improved by LXJDF, we determined the expressions of PCNA, Ki67, loricrin, and LOX-1 in mice skin using immunofluorescence staining techniques. The control group showed the expression of loricrin (red), which formed a continuous line in the keratinization layer. A small number of PCNA (green) and Ki67 (purple) were expressed and slightly overlapped in the basal layer. There are a few expressions of LOX-1 (gold) in the dermis. In the skin of IMQ-induced ApoE^−/−^ mice, loricrin is expressed in both the keratinization layer and exfoliated scales and showed discontinuous lines. The expressions of PCNA and Ki67 increased significantly, and the positive expression of LOX-1 could be seen in the epidermis and dermis. LXJDF could increase the expression of loricrin and inhibited the expressions of PCNA, Ki67, and LOX-1, especially PCNA. Atorvastatin showed apparent inhibition of the expressions of PCNA and LOX-1 (Figure 4C).

We also observed the inflammatory infiltration in each group, including T cells (CD3; CD4), macrophages (F4/80), and dendritic cells (CD11c). There were a small number of expressions of CD4 (green), CD11c (purple), and F4/80 (gold) in the dermis of the control group. IMQ-induced ApoE^−/−^ mice showed a large number of positive expressions of CD3, CD4, F4/80, and CD11c. LXJDF-H could slightly inhibit the expression of CD3 and LXJDF-L could reduce the expressions of CD4 and F4/80. Atorvastatin reduced the expressions of CD3, CD4, CD11c, and F4/80 significantly (Figure 4D).

### Effects of LXJDF on Lipid Level and Hepatic Pathology in IMQ-Induced ApoE^−/−^ Mice

**FIGURE 5:**
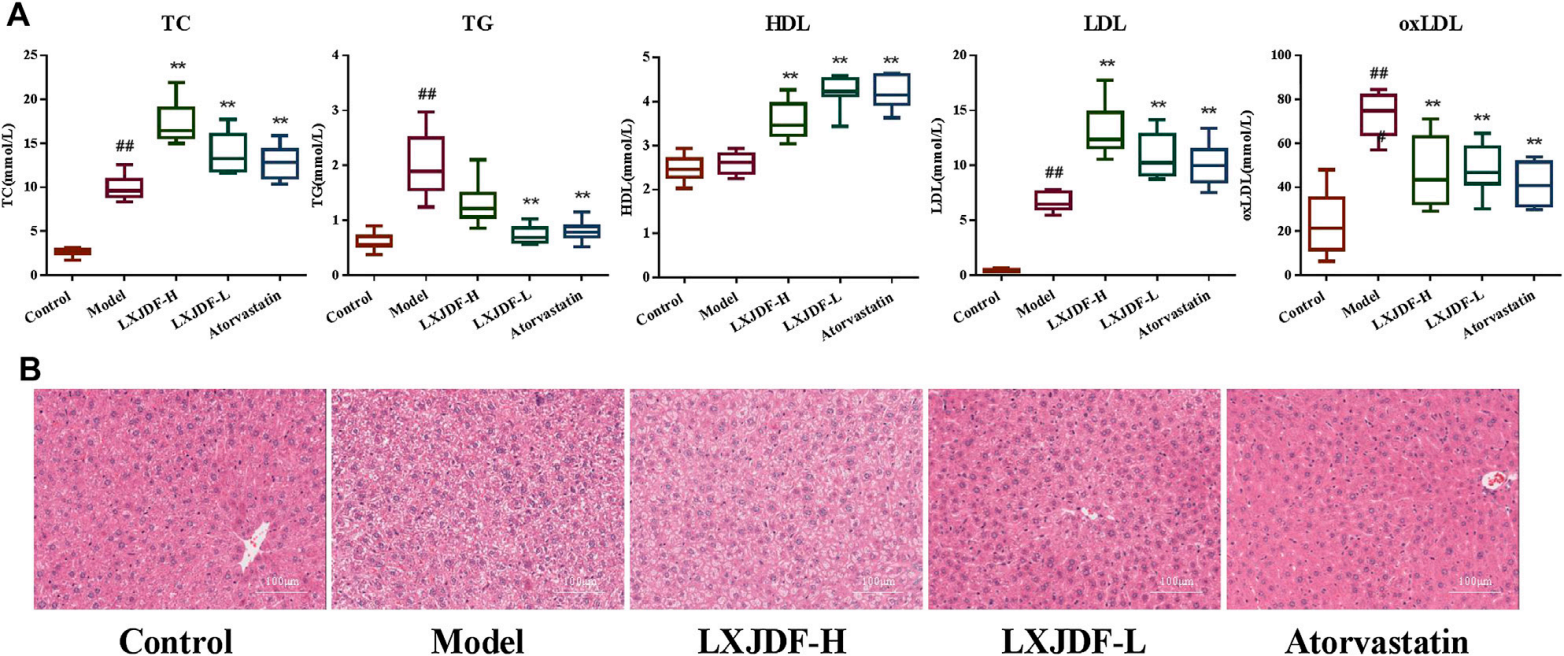
Effects of LXJDF on lipid level and hepatic pathology in IMQ-induced ApoE^−/−^ mice. **(A)** Blood lipid total cholesterol (TC), triglyceride (TG), low-density lipoprotein (LDL), high-density lipoprotein (HDL), and oxidized low-density lipoprotein (oxLDL) levels in the control, model, LXJDF-H, LXJDF-L, and atorvastatin groups (*n* = 10). Results are shown as the mean ± SEM. ^#^ *p* < 0.05 and ^##^ *p* < 0.01 vs. control group; *p < 0.05 and **p < 0.01 vs. model group. **(B)** Hematoxylin and eosin (H&E) staining of the liver in control, model, LXJDF-H, LXJDF-L, and atorvastatin groups. Scale bar = 100 μm.

The levels of TC, TG, LDL, and oxLDL in the model group were significantly higher than the levels of those in the control group, but there was no difference in HDL. LXJDF could reduce TG and oxLDL, and increase HDL, TC, and LDL. It appeared to be consistent with the pharmacodynamic effect of atorvastatin (Figure 5A). Hepatic histology stain showed that the control group’s hepatocytes were neatly arranged and dense, with a few Kupffer cells. In the model group, there were a large number of lipid droplet vacuoles, destroyed or necrotic hepatocytes, and increased Kupffer cells. LXJDF significantly reduced the lipid vacuoles and the structure of hepatocytes was relative integrity. Atorvastatin also reduced lipid vacuoles and improved hepatic injury (Figure 5B).

### Effects of LXJDF on Inflammatory Cytokine Gene Expressions in IMQ-Induced ApoE^−/−^ Mice

**FIGURE 6:**
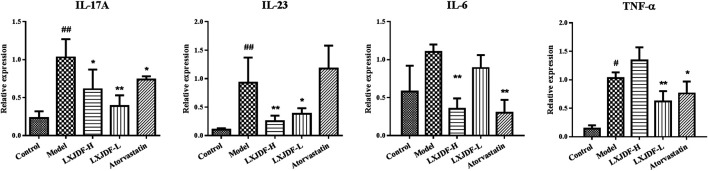
Effects of LXJDF on inflammatory cytokine gene expressions in IMQ-induced ApoE^−/−^ mice. Real-time PCR analysis of the relative levels of IL-17A, IL-23, IL-6, and TNF-α mRNA expressions in the control, model, LXJDF-H, LXJDF-L, and atorvastatin groups (*n* = 4). Results are shown as the mean ± SEM. ^#^ *p* < 0.05 and ^##^ *p* < 0.01 vs. control group; ^*^ *p* < 0.05 and ^**^ *p* < 0.01 vs. model group.

Compared with the control group, the inflammatory factors IL-17A, IL-23, IL-6, and TNF-α mRNA expressions of the model mice skin upregulated significantly. LXJDF-H inhibited the expressions of IL-17A, IL-23, and IL-6, and LXJDF-L had inhibition on the expressions of IL-17A, IL-23, and TNF-α significantly. Atorvastatin inhibited the expressions of IL-17A, IL-6, and TNF-α significantly, as seen in Figure 6, which shows the effects of LXJDF on PI3K/Akt/mTOR protein expressions in IMQ-induced ApoE^−/−^ mice skin.

**FIGURE 7:**
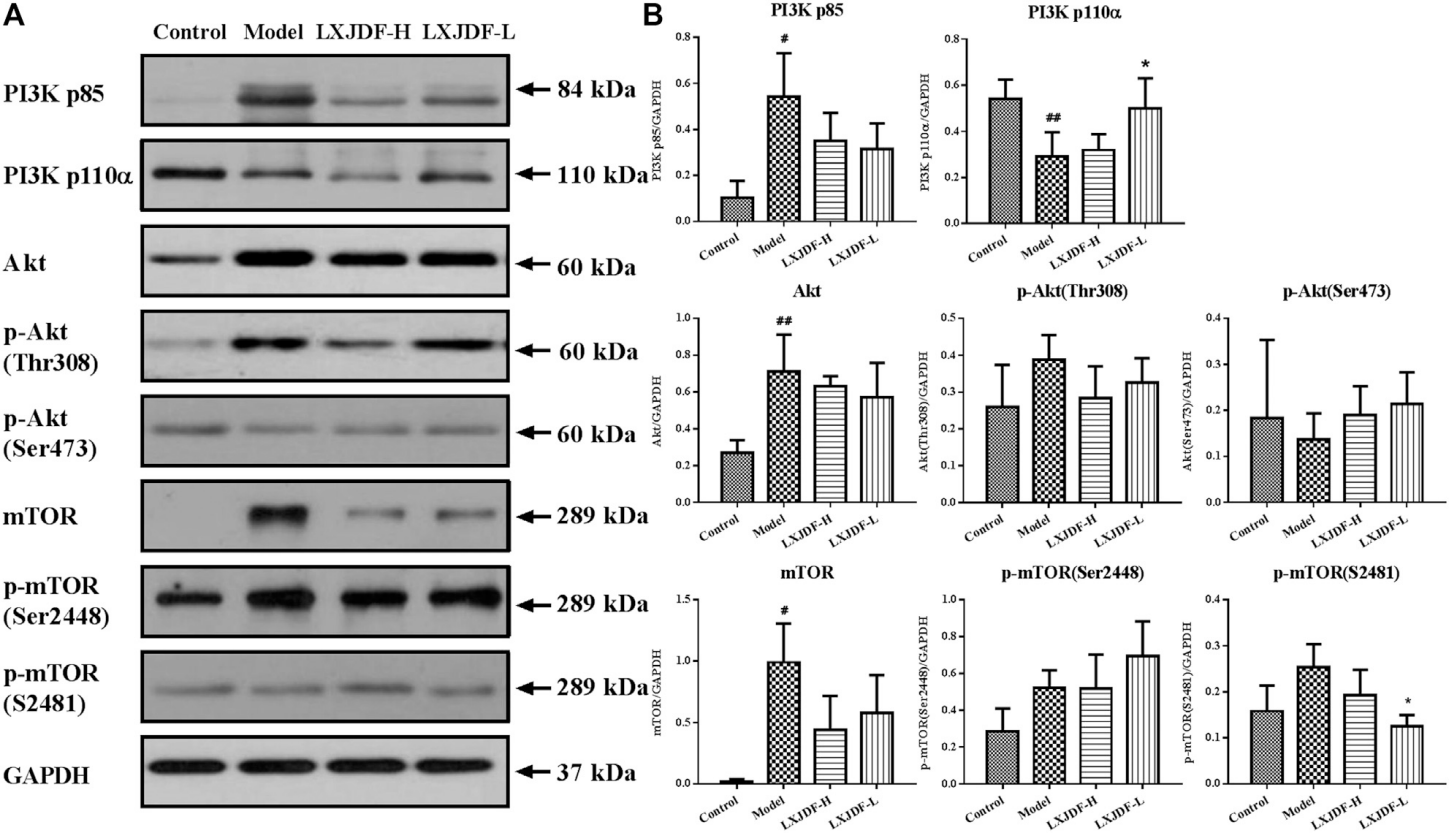
Effects of LXJDF on PI3K/Akt/mTOR protein expressions in IMQ-induced ApoE^−/−^ mice skin. **(A)** Representative images of Western blot showing PI3K p85, PI3K p110α, Akt, p-Akt (Thr308), p-Akt (Ser473), mTOR, p-mTOR (Ser2448), and p-mTOR (S2481) expression in the skin of control, model, LXJDF-H, and LXJDF-L groups. GAPDH was used to confirm an equal amount of protein (*n* = 3). **(B)** Quantitation of PI3K p85, PI3K p110α, Akt, p-Akt (Thr308), p-Akt (Ser473), mTOR, p-mTOR (Ser2448), and p-mTOR (S2481) expression in different groups. Results are shown as the mean ± SEM. ^#^ *p* < 0.05 and ^##^ *p* < 0.01 vs. control group; ^*^ *p* < 0.05 and ^**^ *p* < 0.01 vs. model group.

The total protein expressions of PI3K p85, Akt, and mTOR in the skin lesions of IMQ-induced ApoE^−/−^ mice were significantly increased, while the protein expression of PI3K p110α was significantly decreased. LXJDF could suppress the protein expressions of PI3K p85, mTOR, and p-mTOR (S2481). LXJDF-L could decrease p-mTOR (S2481) and increase PI3K p110α significantly. LXJDF had no significant effect on Akt and p-mTOR (Ser2448) (Figure 7).

Effects of LXJDF on PI3K/Akt/mTOR protein expressions in IMQ-induced ApoE^−/−^ mice liver.

**FIGURE 8:**
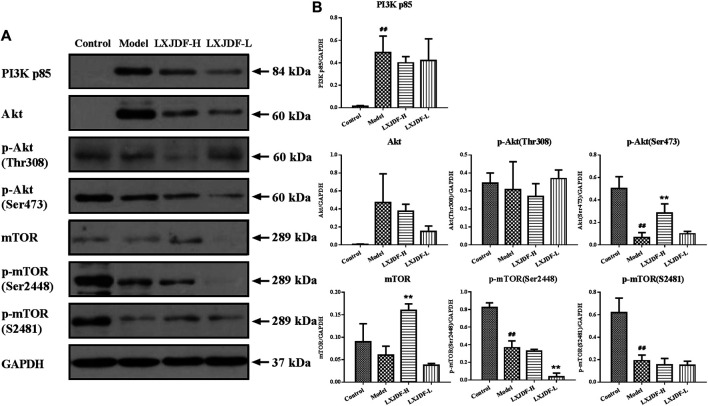
Effects of LXJDF on PI3K/Akt/mTOR protein expressions in IMQ-induced ApoE^−/−^ mice liver. **(A)** Representative images of Western blot showing PI3K p85, Akt, p-Akt (Thr308), p-Akt (Ser473), mTOR, p-mTOR (Ser2448), and p-mTOR (S2481) expression in the liver of control, model, LXJDF-H, and LXJDF-L groups. GAPDH was used to confirm an equal amount of protein (*n* = 3). **(B)** Quantitation of PI3K p85, Akt, p-Akt (Thr308), p-Akt (Ser473), mTOR, p-mTOR (Ser2448), and p-mTOR (S2481) expression in different groups. Results are shown as the mean ± SEM. ^#^ *p* < 0.05 and ^##^ *p* < 0.01 vs. control group; ^*^ *p* < 0.05 and ^**^ *p* < 0.01 vs. model group.

The protein expressions of PI3K p85 and Akt in the liver of mice in the model group increased, and the protein expressions of p-Akt (Ser473), p-mTOR (Ser2448), and p-mTOR (S2481) decreased. LXJDF could suppress Akt and p-mTOR (Ser2448) protein expression and increase p-Akt (Ser473) and mTOR protein expressions. PI3K p110α was not detected in mice liver (Figure 8).

## Discussion

Psoriasis is an inflammatory immune disease accompanied by metabolic disorders and dyslipidemia that can also induce a chronic inflammation response. The PI3K/Akt/mTOR pathway is one of the meaningful pathways regulating inflammation response and organic metabolism. The present article confirmed that LXJDF could regulate the PI3K/Akt/mTOR pathway to intervene in psoriasis and dyslipidemia comorbidity by the experimental study of LXJDF on the IMQ-induced ApoE^−/−^ mice model.

LXJDF could significantly ease IMQ-induced psoriasiform skin lesions embodied by the reduction of scaling, erythema, and infiltration, in addition to inhibiting epidermal hyperplasia (PCNA and Ki67), parakeratosis (loricrin), dermal inflammatory infiltration (CD3, CD4, F4/80, and CD11c), and epidermal lipid accumulation (LOX-1). We determined the expressions of PCNA and Ki67 in the epidermis basal layer at the same for observing keratinocyte proliferation. PCNA is a nucleoprotein required for DNA replication in eukaryotic cells. It gradually increases in the G1 phase, reaches a peak in the S phase, and decreases in the G2/M phase reflecting cell proliferation activity (Schonenberger et al., 2015). Ki67 exists in all active phases of the cell cycle (G1, S, G2, and M phase), but it is absent in resting cells (G_0_ phase) (Bruno and Darzynkiewicz, 1992). The protein expression of Ki67 increases significantly during S phase cells (Darzynkiewicz et al., 2015). The results revealed the co-localization of PCNA and Ki67 protein and marked that the basal cells were in the different phases of mitosis. The distribution of loricrin decreases in skin lesions and non-lesional skin of psoriasis patients (Kim et al., 2011). The results showed that the loricrin expression in the epidermis of the model group reduced, and LXJDF had an improvement effect. We reported that LOX-1 expression was observed in the whole epidermis and part of the dermis layer in IMQ-induced ApoE^−/−^ mice (Xie et al., 2017). LXJDF could significantly inhibit the expression of LOX-1 and reduce lipid accumulation in the skin. Atorvastatin, the positive drug, which is the mainstay of treatment for hyperlipidemia, has been reported to reduce psoriasis risk for short-term statins treatment (Brauchli et al., 2011; Ports et al., 2017). In this experiment we also observed the improvement effect of Atorvastatin on psoriatic dermatitis.

LXJDF could inhibit inflammatory cell infiltration in the dermal layer of IMQ-induced ApoE^−/−^ mice and have a certain inhibition on the gene expressions of proinflammatory factors IL-17A, IL-23, IL-6, and TNF-α. Immune cells and the related cytokines of skin lesions are the crucial pathogenic factor in psoriasis (Sajja et al., 2018). The adipose tissue of psoriasis patients also contains immune cells such as T cells, DCs, neutrophils, mast cells, and adipose tissue macrophages (ATM) the pathological basis for the development of psoriasis into CVD (Rose et al., 2014). The dermis of model mice showed a large number of inflammatory infiltrations, including T cells (CD3, CD4), macrophages (F4/80), and dendritic cells (CD11c). The IL-23/Th17 axis plays a central role in psoriasis' pathogenesis (Van der Fits et al., 2009). We confirmed IL-23/Th17 axis activation in IMQ-induced ApoE^−/−^ mice skin lesions, which is consistent with the immune pathogenesis of IMQ-induced wild-type mice (Xie et al., 2017). Rising inflammatory factors such as IL-17A may promote an increase in intracellular cholesterol and then inflow circulation, leading to a parallel rise in blood cholesterol levels, which may be one of the reasons for psoriasis and dyslipidemia comorbidity (Varshney et al., 2016). Statins are beneficial to psoriasis treatment, possibly by inhibiting the IL-17A signaling pathway (Shirinsky and Shirinsky, 2007). Atorvastatin used as an adjuvant therapy with currently existing standard therapy (topical betamethasone) in patients having mild-to-moderate plaque-type psoriasis modifies the immune function and inhibits inflammatory process and thus protects them against cardiovascular risk (Asad et al., 2017). LXJDF could significantly reduce the inflammatory infiltration of T cells, macrophages, and dendritic cells and repress skin inflammation deterioration.

In this experiment, LXJDF decreased TG and oxLDL and increased HDL, which was inconsistent with Atorvastatin. The elevated TC, TG, and LDL of ApoE^−/−^ mice reduced under the action of IMQ but were still higher than the control group, showing a phenotype of dyslipidemia (Xie et al., 2017). We observed the effect of LXJDF on reducing TG after four consecutive weeks of administration. Psoriasis is related to reactive oxygen species (ROS), and ROS production will reduce the body’s antioxidant abilities. Oxygen metabolites promote the formation of atherosclerotic plaques and causes the infiltration of inflammatory cells by modifying proteins and lipids such as oxLDL (Wakkee et al., 2007). As a marker of dyslipidemia and early atherosclerosis, oxLDL is deemed a biomarker of CVD risk in psoriasis patients. Application of lipid testing techniques has proved a more atherogenic lipid profile and decreased HDL cholesterol efflux capacity (CEC) among patients with vs. without psoriasis, beyond CVD risk factors (Tom et al., 2016). Therefore, LXJDF could prevent the development of dyslipidemia to atherosclerosis.

The administration of LXJDF caused an increase in TC and LDL levels might be related to inhibiting inflammation and regulating cholesterol synthesis and secretion in the liver. Applying IMQ on BALB/c mice for 7 days confirmed that psoriatic inflammation leads to hepatic inflammation, which results in dysregulated protein/lipid metabolism through IL-17RC/NF-КB signaling. It might be one reason for the increased risk of CVD in psoriasis patients (Al-Harbi et al., 2017). Cholesterol synthesis or secretion by the liver may be downregulated due to inflammation as it is an important biomolecule required for several critical biosynthetic reactions such as bile acids and steroid hormones. Hypoalbuminemia occurs when there is increased inflammation, which indicates that liver inflammation caused by psoriasis leads to disordered protein synthesis (Arroyo et al., 2014). Long-term chronic psoriasis inflammation can result in hepatic injury. After applying IMQ for 9 weeks, mice with dermatitis displayed hepatitis, as shown by elevation of plasma transaminase levels and portal and periportal hepatitis, characterized by T-lymphocyte and polymorphonuclear cell infiltrates. This becomes more serious when hepatitis progresses towards liver fibrogenesis (Vasseur et al., 2018). In this experiment, IMQ-induced ApoE^−/−^ mice liver cell structure was destroyed and lipid droplet reduction. LXJDF had the inhibition of liver inflammation and protected the liver.

The PI3K/Akt/mTOR pathway plays a vital role in various biological activities, including innate immunity and body metabolism. PI3K is composed of an 85 kDa regulatory subunit and a 110 kDa catalytic subunit. In psoriasis, PI3K activation triggers the phosphorylation of a 3-hydroxyl group in psoriasis, which then activates Akt kinase through phosphorylation of Thr308 and Ser473, promotes keratinocytes hyperproliferation, and inhibits differentiation (Huang et al., 2014). Akt is highly activated in the epidermal layers of psoriatic lesions. Under the presence of PI-dependent protein kinase, Akt promotes cell proliferation in the epidermis (Madonna et al., 2012). Akt phosphorylation activates a series of proteins, including the mTOR signaling pathway, which can be strictly regulated by a feedback loop. mTOR has two functionally distinct protein complexes (mTORC1 and mTORC2). mTORC2 phosphorylates Akt Ser473 to regulate proliferation and cell growth (Sarbassov et al., 2005). The upregulation of PI3K/Akt and mTOR kinases had been verified in the skin lesions of psoriasis patients and IMQ-induced murine skin. Furthermore, the dual blocking of PI3K/Akt and mTOR signaling pathways could improve IMQ-induced psoriasiform skin lesions in mice (Chamcheu et al., 2016; Chamcheu et al., 2017). During the process of IL-17A-induced inflammatory response in keratinocytes, the activation of the PI3K/Akt/mTOR pathway promotes cholesterol increased, and both could inhibit autophagy and accelerate inflammation (Varshney and Saini, 2018). The protein expressions of PI3K p85, Akt, and mTOR in the skin lesions of IMQ-induced ApoE^−/−^ mice were significantly increased. LXJDF could downregulate the PI3K/Akt/mTOR pathway to improve psoriasiform skin lesions and inhibit the inflammatory response.

In the liver, the activation of mTORC1 predominantly relies on nutrients (amino acids, glucose, nucleotides, fatty acids, and lipids) and growth factors. Nutrients facilitate the translocation of mTORC1 from the cytoplasm to the lysosomal surface, regulating protein synthesis, glucose, and lipid metabolism by the PI3K/Akt signaling (Lu et al., 2020). Continuous activation of mTORC1 does not lead to lipid accumulation due to the potent inhibition of Akt as part of a mTORC1 feedback mechanism. Moreover, given that mTORC2 activates Akt, mTORC2 is also involved in the process of lipogenesis. Blocking the mTORC1 signaling pathway can also increase LDL-C by downregulating liver LDL receptors (Kurdi et al., 2018). The expressions of mTOR and phosphorylate-mTOR protein in the liver of IMQ-induced ApoE^−/−^ mice were significantly reduced. So it was speculated that the strong immune activation of IMQ reduced the liver fatty acids synthesis and lipid levels, and the inhibition of pathway proteins was caused by metabolic disorders. The decrease of p-Akt (Ser473) might be related to the feedback inhibition of mTOR. From the results, we have seen different regulatory effects of LXJDF on PI3K, Akt, and mTOR protein. It might be due to the effect of the LXJDF confront with the liver inflammation caused by IMQ, which involves more complex inflammatory factors and metabolites, and need to be further explored. Another reason might be related to the short period of administration (4 weeks). Therefore, LXJDF could regulate part of the PI3K/Akt/mTOR pathway, reduce TG and oxLDL, and increase HDL to adjust lipid metabolism disorders.

## Conclusion

**FIGURE 9:**
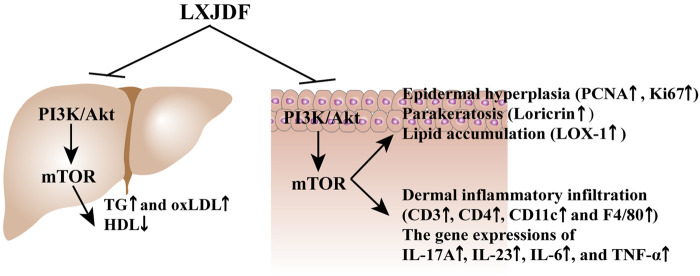
LXJDF improve psoriasis and dyslipidemia comorbidity via PI3K/Akt/mTOR pathway.

Liangxue Jiedu formula (LXJDF) can improve psoriasis skin lesions and regulate lipid metabolism. It has a positive effect, improving the treatment of psoriasis and dyslipidemia comorbidity through regulation of PI3K/Akt/mTOR and its phosphorylation pathway (Figure 9).

## Data Availability

The original contributions presented in the study are included in the article/Supplementary Material; further inquiries can be directed to the corresponding authors.
